# Supplementary material for: Thrombin Is an Effective and Safe Therapy in the Management of Bleeding Gastric Varices. A Real-World Experience
Source: J Clin Med. 2021 Feb 16;10(4):785. doi: 10.3390/jcm10040785 (PMC7920079; doi:10.3390/jcm10040785)
Supplement: Supplementary file 1 [file jcm-10-00785-s001.pdf]

Table S1: Admission platelet count (all groups)

**Patient group**		**Platelet count**<br>Median (range ± SD)
Cirrhosis	Child-Pugh A	118 (53–322, 62.1)
	Child-Pugh B	110 (24–582, 90.2)
	Child-Pugh C	98 (42–491, 88.2)
Non-cirrhotic portal HTN		174 (46–407, 119.1)
No evidence portal HTN (gastric cancer) (*n* = 1)		349 (n/a)

Gastric varices	107 (24–582, 102.8)
Oesophageal varices	87 (33–327, 85.8)
EBL-induced ulceration	117 (47–176, 31.5)
Ectopic varices	146 (42–335, 92.2)
